# Hip Fracture as a Systemic Disease in Older Adults: A Narrative Review on Multisystem Implications and Management

**DOI:** 10.3390/medsci13030089

**Published:** 2025-07-11

**Authors:** Silvia Andaloro, Stefano Cacciatore, Antonella Risoli, Rocco Maria Comodo, Vincenzo Brancaccio, Riccardo Calvani, Simone Giusti, Mathias Schlögl, Emanuela D’Angelo, Matteo Tosato, Francesco Landi, Emanuele Marzetti

**Affiliations:** 1 Department of Translational Medicine and Surgery, Università Cattolica del Sacro Cuore, Largo F. Vito 1, 00168 Rome, Italy; silvia.andaloro01@icatt.it (S.A.); vincenzo.brancaccio01@icatt.it (V.B.); 2 Department of Geriatrics, Orthopedics and Rheumatology, Università Cattolica del Sacro Cuore, Largo F. Vito 1, 00168 Rome, Italy; antonella.risoli01@icatt.it (A.R.); roccomaria.comodo01@icatt.it (R.M.C.); riccardo.calvani@unicatt.it (R.C.); francesco.landi@unicatt.it (F.L.);; 3 Fondazione Policlinico Universitario “Agostino Gemelli” IRCCS, Largo A. Gemelli 8, 00168 Rome, Italy; simone.giusti@guest.policlinicogemelli.it (S.G.); emanuela.dangelo@policlinicogemelli.it (E.D.); 4 Department of Orthopaedics, Mater Olbia Hospital, Strada Statale 125 Orientale Sarda, 07026 Olbia, Italy; 5 Research Unit of Orthopaedic and Trauma Surgery, Department of Medicine and Surgery, Università Campus Bio-Medico di Roma, Via Álvaro del Portillo, 21, 00128 Rome, Italy; 6 Division of Geriatric Medicine, Clinic Barmelweid, 5017 Barmelweid, Switzerland; mathias.schloegl@barmelweid.ch

**Keywords:** frailty, sarcopenia, intrinsic capacity, osteoporosis, osteosarcopenia, rehabilitation, orthogeriatric care, biomarkers, biological aging

## Abstract

Hip fractures are among the most serious health events in older adults, frequently leading to disability, loss of independence, and elevated mortality. In 2019, an estimated 9.6 million new cases occurred globally among adults aged ≥ 55 years, with an incidence rate of 681 per 100,000. Despite improved surgical care, one-year mortality remains high (15–30%), and fewer than half of survivors regain their pre-fracture functional status. Traditionally regarded as mechanical injuries, hip fractures are now increasingly recognized as systemic events reflecting and accelerating biological vulnerability and frailty progression. We synthesize evidence across biological, clinical, and social domains to explore the systemic implications of hip fracture, from the acute catabolic response and immune dysfunction to long-term functional decline. The concept of intrinsic capacity, introduced by the World Health Organization, offers a resilience-based framework to assess the multidimensional impact of hip fracture on physical, cognitive, and psychological function. We highlight the importance of orthogeriatric co-management, early surgical intervention, and integrated rehabilitation strategies tailored to the individual’s functional reserves and personal goals. Innovations such as digital health tools, biological aging biomarkers, and personalized surgical approaches represent promising avenues to enhance recovery and autonomy. Ultimately, we advocate for a shift toward interdisciplinary, capacity-oriented models of care that align with the goals of healthy aging and enable recovery that transcends survival, focusing instead on restoring function and quality of life.

## 1. Introduction

Hip fractures are among the most severe age-related injuries, carrying significant clinical and public health implications. They are a leading cause of disability, institutionalization, and mortality in older adults, often resulting in irreversible loss of autonomy and quality of life. Over the past three decades, their global incidence has risen sharply [1]. In 2019, an estimated 9.6 million new cases occurred among adults aged 55 and older, with an incidence rate of 681 per 100,000 [2]. While women remain more frequently affected, the rate of increase has been faster among men. Overall, nearly 17 million individuals were living with the consequences of a hip fracture, which collectively accounted for more than 1.8 million years lived with disability [2]. Despite advances in surgical and perioperative management, one-year mortality rates remain high, typically ranging from 15% to 30% [1], and fewer than half of survivors regain their pre-fracture functional status [3].

Hip fractures in older adults are no longer seen merely as mechanical injuries requiring surgical repair but are increasingly recognized as systemic events occurring in clinically vulnerable individuals [4]. Moreover, the trauma of the fracture and subsequent immobility initiate a complex cascade involving systemic inflammation, metabolic disruption, and multisystem decompensation [5,6]. These changes often precipitate acute complications such as delirium, pneumonia, venous thromboembolism, and cardiovascular events, and contribute to the progression of sarcopenia and frailty [7,8]. The conceptual framing of hip fracture as a systemic condition reflects a broader paradigm shift in geriatric medicine, from a disease-centered to a capacity-oriented paradigm [9]. In this view, hip fracture is not merely the consequence of osteoporosis or an accidental fall, but the result of a broader interplay between musculoskeletal decline, immune senescence, neuroendocrine dysfunction, and increased vulnerability. Conditions such as osteosarcopenia, marked by the concurrent loss of bone and muscle mass, highlight the synergistic nature of musculoskeletal aging and its systemic reverberations [10]. Recovery is further influenced by frailty status and its associated factors, including multimorbidity, cognitive decline, prolonged immobilization, poor nutritional status, and unfavorable health self-perception [11,12]. These factors support the growing emphasis on orthogeriatric care, an integrated, guideline-endorsed model that combines timely surgery with comprehensive geriatric assessment, interdisciplinary management, and personalized rehabilitation. While increasingly implemented, its adoption remains variable across healthcare systems [13].

This narrative review proposes a reframing of hip fracture as a systemic disease in older adults by exploring its pathophysiological impact across multiple organ systems. We synthesize evidence from biological, clinical, and social domains, emphasizing the interconnected mechanisms that amplify vulnerability and compromise recovery. By adopting a multisystem perspective, we advocate for an interdisciplinary, resilience-oriented approach to care, ultimately aimed at guiding more effective, biologically informed, and patient-centered management strategies.

## 2. Epidemiology and Risk Factors

### 2.1. Global and Regional Incidence and Mortality Trends

Hip fractures represent a growing global health concern, driven primarily by population aging and the increasing prevalence of frailty-related conditions [12]. Despite regional differences in age-standardized rates, this steep rise reflects a global trend toward longer life expectancy and a greater burden of chronic diseases that compromise bone and muscle integrity [2]. Marked geographic variability highlights the complex interplay between demographic, environmental, and healthcare system-related factors. For example, Denmark reports the highest incidence globally, with 315.9 cases per 100,000 individuals, while Brazil records a significantly lower rate of 95.1 per 100,000 [1]. These differences likely reflect underlying disparities in healthcare infrastructure and public health strategies, including the availability of diagnostic tools, access to osteoporosis treatment, nutritional patterns affecting bone health, and the implementation of falls prevention programs. In the United Kingdom, although age-adjusted incidence has declined, possibly due to improved osteoporosis screening and preventive interventions, the absolute number of fractures is expected to double within the next 25 years due to demographic aging [1].

Large epidemiological studies consistently report that women account for approximately 70% of hip fracture cases. A global analysis of over 4 million hip fractures demonstrated that the majority of cases occur in women, reflecting both higher osteoporosis prevalence and longer life expectancy in women [1]. However, men experience worse outcomes after hip fracture, including up to twofold higher post-fracture mortality, and slower or less complete functional recovery [14,15,16].

Mortality following hip fracture remains unacceptably high. The rates of one-year mortality for all causes range from 14.4% to 28.3%, with consistently higher rates observed in men [1]. Functional recovery is frequently incomplete, with many patients experiencing prolonged dependence, institutionalization, or readmission [3,17,18]. These epidemiologic patterns underscore the urgent need for preventive strategies and systemic models of care.

### 2.2. Risk Factors: Biological, Environmental, and Social Determinants

**Figure 1:**
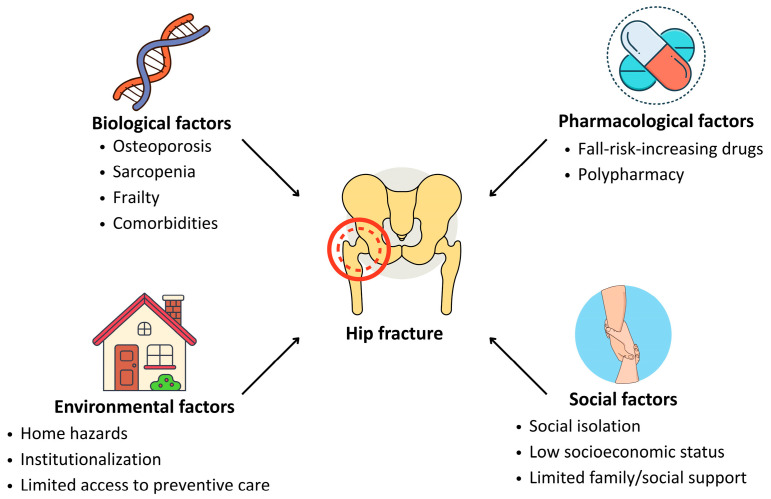
Main risk determinants for hip fracture in older adults.

Hip fractures result from the convergence of multiple interdependent factors that compromise skeletal resilience, neuromuscular function, and behavioral safety. These can be broadly categorized into biological, pharmacological, environmental, and social determinants (Figure 1).

Aging is associated with progressive loss of bone mineral density, impaired balance, slowed protective reflexes, and an increased burden of chronic disease [19]. Osteoporosis remains a central contributor to fracture risk, resulting from both non-modifiable factors (e.g., sex, genetics, endocrine changes) and modifiable ones, such as calcium and vitamin D deficiency, sedentary behavior, and long-term corticosteroid use [20]. Sarcopenia, often coexisting with osteoporosis in the syndrome of osteosarcopenia, increases the likelihood of falls and reduces the capacity for protective postural responses [21]. In community-dwelling adults aged 65 years and older, the prevalence of osteosarcopenia is estimated to range from 5% to 37%, with substantially higher rates (17–96%) observed in individuals with prior fractures. Notably, osteosarcopenia is associated with nearly fourfold higher odds of fracture [21]. Importantly, up to 90% of hip fractures occur after low-energy trauma, such as slipping or minor missteps, indicating that intrinsic musculoskeletal vulnerability outweighs the force of the fall [21,22]. Neurological and cardiovascular conditions also increase fracture risk. For instance, stroke patients, due to hemiparesis and immobility, experience accelerated bone loss and postural instability [23]. Likewise, individuals with heart failure, ischemic heart disease, or peripheral artery disease exhibit fracture rates several times higher than those without such comorbidities, possibly reflecting impaired perfusion, reduced mobility, and systemic inflammation [23,24,25]. Endocrine and metabolic disorders, including hyperthyroidism, diabetes, and low body weight (BMI < 22), further exacerbate bone fragility [26,27,28], while female reproductive factors such as early menopause or menstrual irregularities contribute to reduced estrogen exposure and diminished bone mass [29].

Polypharmacy and the use of fall-risk-increasing drugs (FRIDs), notably benzodiazepines, antidepressants, and antihypertensives, are prevalent among older adults and independently associated with falls and fractures [30,31]. According to Correa-Pérez et al. [31], patients discharged after hip fracture were prescribed an average of nearly 12 medications, with a mean of 2.9 classified as FRIDs. Substance use, particularly excessive alcohol intake and smoking, accelerates bone loss and impairs neuromuscular control, thus compounding fracture risk [32]. Living in long-term care facilities is associated with a markedly elevated incidence of hip fractures, likely due to environmental hazards, nutritional deficiencies, and reduced mobility [33,34]. In contrast, adherence to a Mediterranean diet and regular physical activity have been identified as protective factors [35,36].

Socioeconomic status profoundly shapes fracture risk and recovery potential. Limited financial resources, low educational attainment, and weak social support networks are associated with both increased incidence and worse post-fracture outcomes [37]. Institutionalization, poor housing conditions, and social isolation contribute to fall risk and delay in accessing timely medical care [37]. Conversely, being married or having close family support has been associated with faster rehabilitation and greater chances of returning to independent living [38]. These findings reinforce the need for comprehensive care models that integrate not only clinical management but also social and environmental interventions.

## 3. Hip Fracture, Frailty, and Systemic Decline in Older Adults

### 3.1. Understanding Frailty in the Context of Hip Fracture

Frailty is a multidimensional clinical syndrome characterized by a decline in physiological reserves and increased vulnerability to stressors, involving alterations in multiple biological systems, including musculoskeletal, immune, endocrine, and cognitive domains [39]. In the setting of hip fracture, frailty is both a predisposing factor and a determinant of outcomes. Frail individuals are disproportionately affected by hip fractures and are more likely to experience complications such as delirium, functional decline, and mortality [40,41,42,43,44]. Rather than a mechanical event alone, hip fracture in frail patients may represent a sentinel event signaling advanced biological vulnerability.

### 3.2. Pathophysiological Mechanisms Linking Frailty and Hip Fracture

Multiple interrelated mechanisms underlie the relationship between frailty and hip fracture. Reduced physical activity, sarcopenia, and malnutrition are key contributors to both bone loss and muscle wasting. Sarcopenia, defined by loss of skeletal muscle mass and strength, is significantly associated with falls and fractures [45]. When compounded by osteoporosis, this syndrome of osteosarcopenia significantly increases fracture risk [46]. Fall frequency, which is significantly associated with frailty and sarcopenia, appears to be a stronger predictor of fracture than bone mineral density (BMD) alone [47]. Observational studies and large cohort analyses, such as the European Prospective Osteoporosis Study, confirm that the highest fracture risk occurs in individuals with both elevated fall risk and low BMD [48]. This highlights the limitations of relying solely on bone density for risk stratification and underscores the importance of addressing functional status.

Nutritional status plays a pivotal role in the frailty–hip fracture axis. Protein energy malnutrition, hypoalbuminemia, and micronutrient deficiencies (particularly vitamin D) impair bone and muscle health [49,50,51,52,53]. Weight loss, whether intentional or unintentional, is a recognized predictor of fracture and mortality. For instance, data from the GLOW study show that a loss of 4.5 kg or more increases fracture risk within a year and for several years thereafter [54]. Similarly, extremes in weight variability, especially in individuals with diabetes, have been linked to elevated hip fracture risk [55].

Endocrine dysregulation further exacerbates this vulnerability. Aging is associated with reduced levels of anabolic hormones such as testosterone, estrogen, insulin-like growth factor 1 (IGF-1), and growth hormone, all of which play critical roles in musculoskeletal maintenance [29,56,57]. IGF-1, primarily induced by GH, plays a crucial role in bone strength by stimulating osteoblast activity and promoting bone formation. Low IGF-1 levels are associated with reduced BMD and increased fracture risk. A Mendelian randomization study of over 350,000 individuals showed that each 1-standard-deviation increase in IGF-1 levels was associated with a significant rise in estimated BMD and a corresponding 6% reduction in fracture risk [57]. Inflammatory mediators, particularly interleukin-6 (IL-6), are elevated in frailty and sarcopenia, correlating with muscle catabolism and bone loss [58].

Cognitive impairment also contributes to falls and fractures [8,12]. Older adults with cognitive frailty exhibit reduced executive function, slower reaction times, and impaired judgment, all of which increase the risk of falling [59,60].

### 3.3. Hip Fracture as a Catalyst for Systemic Deterioration

While many diseases in older adults may exert systemic clinical effects, hip fracture, being an acute and profoundly debilitating event, serves as the paradigmatic example of how an acute stressor can unmask underlying pre-existing biological frailty (advanced age, sarcopenia, malnutrition, multiple comorbidities, pre-existing disability) and challenge the body’s stress response mechanisms [61,62]. Hip fractures not only reflect frailty but also accelerate systemic decline through a cascade of pathophysiological events. Surgical stress, bed rest, and inflammation amplify pre-existing deficits and generate new complications [63]. The acute phase response activates the hypothalamic–pituitary–adrenal axis and upregulates catabolic cytokines (e.g., IL-6, TNF-α), further promoting muscle wasting, insulin resistance, and immune dysfunction [64]. Immunosenescence, characterized by impaired T-cell function, reduced neutrophil chemotaxis, and dysregulated macrophage activity, compromises the ability to resolve inflammation and predisposes to infections, delayed healing, and poor outcomes [65]. Disuse atrophy during immobilization profoundly impacts both muscle and bone. Mechanotransduction pathways such as Wnt/β-catenin are suppressed, leading to decreased osteoblast activity and increased osteoclastogenesis [66]. Myostatin levels rise, while IGF-1 and satellite cell activity decline, creating a catabolic state that impairs recovery [67]. These processes intersect to form a “frailty loop,” wherein malnutrition, cognitive decline, sarcopenia, and inflammation reinforce each other in a vicious cycle. This feedback loop contributes to long-term disability, recurrent hospitalizations, and increased mortality.

Recent advances in geroscience suggest that events such as hip fractures may act as acute accelerators of biological aging. A study by Tarpada et al. [68] investigated DNA methylation age in geriatric hip fracture patients. The study found that both blood- and bone-derived DNA methylation ages were significantly higher in patients who died within a year post-fracture, averaging approximately 17 years older than their chronological age. Another study reported that markers of cellular senescence, specifically components of the senescence-associated secretory phenotype, remained elevated in patients up to 12 weeks following hip fracture surgery [69]. This persistent elevation indicates a prolonged catabolic and inflammatory state, potentially accelerating biological aging. These findings underscore the potential of molecular aging clocks to assess biological age and monitor recovery trajectories in older adults post-hip fracture. Identifying patients at risk of accelerated decline could inform targeted interventions aimed at mitigating systemic deterioration and improving outcomes.

### 3.4. Clinical Implications: Stratifying Risk and Personalizing Care

Given the complex interplay between frailty and hip fracture, management strategies must be individualized. Comprehensive geriatric assessment aids in identifying high-risk patients and tailoring perioperative care [13,40]. Surgical intervention remains the mainstay of treatment, but must be approached with caution in frail patients. Timing is critical: procedures performed within 24–48 h are associated with improved outcomes, though ultra-early surgery (<12 h) may carry additional risks in unstable patients [70].

The orthogeriatric care model, characterized by close collaboration between orthopedic surgeons and geriatricians, has been increasingly implemented in several countries [71]. Meta-analyses and large cohort studies consistently show that orthogeriatric care reduces in-hospital mortality (by 28%), 1-year mortality (by 14%), delirium (by 19%), and length of stay (by 1–7 days) compared to standard orthopedic care, with the most pronounced benefits seen in integrated or dedicated orthogeriatric wards. Functional outcomes are variably improved, with some studies showing better mobility and independence at 4 months post-fracture. The benefits are particularly relevant for frail older adults with multimorbidity, sarcopenia, and pre-existing disability, as these patients are at highest risk for poor outcomes after acute stressors like hip fracture [72,73,74,75,76].

The main barriers to the implementation of orthogeriatric models include limited availability of geriatricians, inconsistent use of comprehensive geriatric assessment, and variable adherence to evidence-based protocols for delirium, osteoporosis, and falls prevention. Among organizational barriers, lack of standardized care pathways, poor communication between orthopedic and geriatric teams, and insufficient integration with rehabilitation and community services are frequently reported. Financial barriers include inadequate reimbursement for multidisciplinary care, lack of dedicated funding for orthogeriatric units, and fragmented payment systems that disincentivize integrated care [77,78]. Nevertheless, cost-effectiveness analyses from the U.K. and other European settings demonstrate that orthogeriatric models are either cost-saving or cost-effective, with estimated costs per QALY well below accepted thresholds, largely due to reduced complications, shorter hospitalizations, and lower rates of institutionalization. Annual savings per 1000 patients can reach several million euros, especially when reductions in long-term care needs are included [79,80,81].

## 4. Hip Fracture and Intrinsic Capacity: A Resilience-Based Perspective

In the past decade, the World Health Organization has reformulated its vision of aging, shifting from the concept of active aging to a more comprehensive framework known as healthy aging [82]. At the heart of this shift lies the recognition that the primary goal of care in older age is not simply the absence of disease, but the preservation of functional ability—that is, the capacity to do what matters most to each individual in their daily life [82]. Central to this new perspective is the concept of intrinsic capacity (IC), defined as the overall combination of an individual’s physical and mental abilities [82]. Rather than focusing narrowly on isolated diseases or deficits, IC offers a dynamic and person-centered lens through which to understand the aging process. It places emphasis on resilience, the ability to recover from stressors, as a key determinant of long-term outcomes [83]. Intrinsic capacity encompasses five core domains: locomotion, cognition, vitality, sensory function, and psychological well-being [82]. These domains interact dynamically and together form the foundation of an individual’s ability to adapt to stressors, including illness, injury, or hospitalization [84]. In this view, a hip fracture represents a critical test of IC, as it challenges multiple domains, mobility, cognition, mood, nutrition, and sensory function, bringing to light vulnerabilities that may have been previously compensated [85]. Understanding hip fracture through the lens of IC allows clinicians and researchers to focus not only on survival or symptom control, but on strategies to restore capacity, support adaptation, and preserve autonomy in the face of adversity [86].

The systemic impact of hip fracture manifests as an abrupt deterioration in all IC domains: Locomotion is compromised due to disuse atrophy, surgical trauma, and prolonged immobilization, leading to reduced gait speed, balance, and coordination, key predictors of future falls and mortality [87,88]. Vitality declines as the catabolic response to trauma drives energy depletion, sarcopenia, malnutrition, and inflammatory activation, all of which diminish physiological reserves [6,49,63]. Cognitive and psychological functions are frequently impaired. Postoperative delirium, depression, and fear of falling are prevalent and synergistically exacerbate physical decline, perpetuating the frailty loop [42,89,90].

### Integrating Intrinsic Capacity into Clinical Models of Fracture Care

Incorporating IC into falls prevention and hip fracture care enables a personalized and proactive approach to management. Pre-fracture IC assessment can identify patients at high risk of poor outcomes and guide prehabilitation, nutritional optimization, cognitive support, and social reinforcement. Post-fracture, IC domains can inform individualized rehabilitation plans, prioritizing preserved capacities and targeting areas of vulnerability. Multidomain interventions, addressing mobility, mood, nutrition, and social participation, have shown promise in halting or reversing IC decline [91]. The WHO’s Integrated Care for Older People (ICOPE) framework operationalizes this approach, integrating IC assessment into primary care and geriatric pathways [92]. Its adaptation to trauma settings represents an opportunity to align fracture care with healthy aging goals.

In its latest edition [93], the ICOPE Handbook not only consolidates previous domains such as mobility, cognition, and nutrition but also introduces urinary incontinence as a key factor in the health of older persons. This addition is particularly pertinent to hip fracture care, where bladder management remains an often underrecognized yet clinically significant component, given its association with postoperative urinary retention, infectious complications, delirium, and delayed functional recovery [94].

Current clinical guidelines primarily emphasize timely surgery, falls prevention, and secondary fracture prevention [95,96]. Building on these foundations, we advocate for a complementary approach that integrates the concept of IC and targets multisystem recovery. Incorporating IC into clinical pathways shifts the focus beyond survival and complication rates toward a resilience-oriented, function-centered model of care. Such a paradigm better addresses the complexity of hip fracture in aging populations, aligning management strategies with biological heterogeneity and the overarching goal of promoting healthy and dignified aging.

## 5. Rehabilitation and Functional Recovery: Toward a Continuum of Multidimensional Care

### 5.1. Multidisciplinary Models for Post-Fracture Rehabilitation

Effective recovery after hip fracture depends on more than surgical repair, as it requires the orchestration of a comprehensive, multidisciplinary rehabilitation model tailored to the biological and functional vulnerability of older adults. Orthogeriatric co-management represents the most mature example of integrated care for older adults with hip fracture, combining surgical expertise and geriatric principles to improve outcomes across the continuum of hospitalization [71,97]. This team-based approach allows for early identification and management of perioperative complications, such as delirium, infections, and malnutrition, while also supporting early mobilization, pain control, and discharge planning. Standardized care pathways, including checklists for postoperative monitoring, catheter management, and nutritional and hydration support, are critical components of high-quality care delivery in this population.

### 5.2. Personalized Rehabilitation: Integrating Nutrition, Physical Therapy, and Psychosocial Support

Rehabilitation must begin at admission and continue seamlessly across settings—whether in acute care, subacute facilities, or the patient’s home. While early mobilization is widely endorsed, there is a lack of consensus on structured rehabilitation protocols, especially for frail or cognitively impaired patients [98]. A cornerstone of functional recovery is nutritional adequacy. Older adults often enter hospitalization with pre-existing malnutrition and face further declines due to reduced appetite, cognitive impairment, or delirium. Caloric and protein deficits have been independently associated with longer hospital stays, poorer rehabilitation trajectories, and higher mortality [99].

Psychological and social support is another important pillar. Depression, anxiety, and loss of independence are common emotional sequelae following hip fracture and may impair motivation to participate in rehabilitation. Integrating mental health assessment and support improves engagement and can enhance recovery [100].

From a physical perspective, rehabilitation programs should be fracture- and procedure-specific. Initial exercises may include bed mobility, sit-to-stand transfers, chair rises, and walking with assistive devices. Evidence supports the use of intensive, structured physical therapy even in the early post-operative phase, which has been shown to reduce hospital length of stay and promote faster return to activities of daily living [101,102].

### 5.3. Transitional Care and Post-Acute Rehabilitation: Bridging the Recovery Gap

The transition from hospital to home or a rehabilitation facility represents a critical juncture in the recovery pathway of older adults following hip fracture [103]. A growing body of evidence highlights the importance of transitional care models, which ensure continuity of medical, functional, and psychosocial support after discharge. These models involve coordinated discharge planning, structured communication among care providers, and early follow-up interventions. The goal is to mitigate the “rehabilitation cliff” often experienced by frail older adults once they leave the acute care setting [103].

Evidence underscores the critical role of post-acute transitional services in safeguarding patient safety during the shift from hospital to residential care, helping to reduce medical errors and adverse events that frequently occur during care transitions in older adults with complex needs [104]. In rehabilitation facilities, targeted physical and occupational therapy can continue under supervision, while complications such as malnutrition, delirium, or pressure injuries are actively managed. However, outcomes in these settings are highly variable and often depend on staffing ratios, geriatric expertise, and individualized care plans [105,106].

For patients returning home, home-based rehabilitation programs can be effective, particularly when supported by multidisciplinary outreach teams. A quasi-experimental study evaluated the effectiveness of a family-based care transition program on older adults after hip fracture surgery [107]. The program encompassed structured in-hospital education, post-discharge home visits, and follow-up telephone support. Compared to standard care, participants receiving the intervention demonstrated significant improvements in activities of daily living and health-related quality of life.

Early supported discharge programs, wherein physiotherapists, nurses, and geriatricians conduct home visits, have shown promise in maintaining functional gains and reducing caregiver burden. Importantly, these approaches align with patient preferences for aging in place and avoiding institutionalization.

Barriers to the effective implementation of transitional care interventions include the fragmentation of healthcare services, the absence of sustainable reimbursement mechanisms, and limited integration between hospital-based and community-based providers [106,108]. Addressing these gaps through standardized post-discharge follow-up protocols, actively involving general practitioners and community health services, is essential to support recovery trajectories and reduce the risk of preventable readmissions. Ultimately, optimal rehabilitation following hip fracture depends on the establishment of a seamless continuum of care that transcends institutional boundaries, guided by personalized goals, proactive clinical monitoring, and environments designed to meet the biological and functional needs of each individual.

## 6. Controversies, Knowledge Gaps, and Future Directions: Toward a Personalized and Integrated Paradigm

The evolving landscape of hip fracture care reflects a growing recognition that one-size-fits-all models are inadequate in the face of an aging, heterogeneous population. Recent evidence supports a shift from protocolized treatment algorithms to personalized strategies based on pre-fracture function, comorbidity burden, and frailty status. However, key controversies remain.

A major unresolved issue concerns the optimal surgical strategy. Total hip arthroplasty (THA) offers superior functional outcomes compared to hemiarthroplasty (HA) in active older adults [109], but its benefits in frailer individuals are uncertain due to higher risks of dislocation and perioperative complications. The evidence is limited, and robust criteria to guide patient selection are lacking. Emerging techniques, such as dual mobility cup THA (DMC-THA), show promise in balancing mobility and safety but require further validation [110].

Another open question is the role of biomarkers of biological aging and musculoskeletal reserve, including proteomic risk scores, low IGF-1, serum myostatin, TNF-α, and vitamin D, which may help identify individuals at risk for sarcopenia and poor recovery [111,112,113]. Although preliminary studies suggest these biomarkers may predict recovery trajectories, their clinical application remains unproven, and larger validation studies are needed.

At the same time, innovations continue to reshape the field. Advancements in orthopedic surgical tools and techniques are transforming hip fracture management. The development of more biomechanically robust implants, such as dynamic hip screws and intramedullary nails, has improved fixation stability and reduced reoperation rates in unstable fractures [114]. Intraoperative imaging and computer-assisted navigation now allow for greater accuracy in implant placement, while robotic-assisted surgery is emerging as a promising modality to reduce soft tissue trauma, enhance reproducibility, and support minimally invasive approaches [115]. Although these technologies are not yet widely available in all settings, they underscore the potential for precision surgery in geriatric trauma care, provided that implementation is matched by appropriate patient selection and perioperative support.

As post-acute recovery increasingly shifts beyond the hospital setting, digital health technologies are emerging as promising tools to support functional monitoring and care continuity. A recent systematic review and meta-analysis evaluated the effectiveness of home-based digital health interventions in older adults following hip fracture surgery [116]. Interventions such as telerehabilitation and remote monitoring were associated with significant improvements in functional performance, as measured by the timed up and go test and the short physical performance battery. The Hospital to Home study demonstrated that integrating wearable technologies, such as smartwatches, with mobile health applications enables continuous monitoring of physical activity, early detection of risk events, and the delivery of remote coaching support [117]. Patients utilizing these tools exhibited higher daily step counts and a lower incidence of fall-related readmissions, indicating that real-time functional monitoring may not only enhance patient engagement in recovery but also furnish clinicians with actionable data to optimize rehabilitation strategies.

The future of hip fracture care lies not in incremental improvements to isolated procedures but in the integration of predictive biology, digital health, and person-centered care models that prioritize resilience, autonomy, and long-term function. Addressing current knowledge gaps through large-scale, stratified trials and real-world implementation studies will be essential. Specifically, future research should focus on (i) defining precise selection criteria for surgical strategies based on frailty and biological age, (ii) validating the prognostic utility of aging biomarkers to personalize rehabilitation trajectories, (iii) evaluating the long-term effectiveness and scalability of digital health interventions in diverse clinical settings, and (iv) developing integrated care models that incorporate intrinsic capacity assessment into routine practice. These efforts will be crucial to achieving truly individualized, resilience-oriented care for older adults with hip fracture.

## 7. Limitations

This narrative review is subject to several inherent limitations. The absence of a systematic search strategy introduces the potential for selection bias and limits the reproducibility of our findings. Moreover, without predefined inclusion and exclusion criteria, the synthesis of evidence remains qualitative and interpretative rather than exhaustive. Given the rapidly evolving nature of hip fracture management and geriatric care, some emerging areas of research may not be fully represented. Finally, while we endeavored to adopt an integrative, multisystem perspective, the breadth of the topic inevitably constrained the depth of discussion on specific domains. These limitations should be acknowledged when interpreting the scope and implications of the review, which is intended to offer a conceptual framework for advancing clinical practice rather than prescriptive recommendations.

## 8. Conclusions

Hip fracture in older adults should be understood not as an isolated orthopedic injury but as a manifestation of systemic vulnerability that accelerates biological and functional decline. This review proposes a reframing of hip fracture as a multisystem condition, closely linked to frailty, sarcopenia, and cognitive impairment, and calls for a shift toward integrated, capacity-oriented models of care. Orthogeriatric approaches, grounded in interdisciplinary coordination and early, individualized rehabilitation, represent a key strategy to mitigate the broader impact of these events. Redefining hip fracture in this way is not only clinically sound but essential to supporting autonomy, dignity, and healthy aging in the aftermath of fracture.

## Data Availability

Not applicable. No new data were created or analyzed in this study.
